# Linking environmental factors and gene regulation

**DOI:** 10.7554/eLife.96710

**Published:** 2024-03-18

**Authors:** Signe Penner-Goeke, Elisabeth B Binder

**Affiliations:** 1 Department of Genes and Environment, Max Planck Institute of Psychiatry Munich Germany

**Keywords:** epigenome, methylation, biological embedding, early life adversity, MPRA, epigenetics, Human

## Abstract

A technique called mSTARR-seq sheds light on how DNA methylation may shape responses to external stimuli by altering the activity of sequences that control gene expression.

**Related research article** Johnston RA, Aracena KA, Barreiro LB, Lea AJ, Tung J. 2024. DNA methylation-environment interactions in the human genome. *eLife* **12**:RP89371. doi: 10.7554/eLife.89371.

Gene regulation is a complex process that allows cells to control when and how they express their genes. Genetic variants can influence these mechanisms, often causing issues that lead to disease (Albert and Kruglyak, 2015). But could environmental signals also play a similar role?

This question has fuelled much interest into epigenetics, a field that focuses on a range of molecular mechanisms which modify DNA while leaving the underlying genetic sequence intact. The best-known example, DNA methylation, is crucial for fundamental biological processes such as cell fate decision. This process relies on methyl groups being added to certain genetic sites. These ‘marks’ can persist through division and therefore be transmitted across cell generations and importantly, environmental factors can influence them (Moore et al., 2013). As such, DNA methylation represents a potential mechanism by which the environment can shape gene expression and subsequent health outcomes. Indeed, early changes in DNA methylation have been proposed to dictate how an organism responds to stressors later in life, enabling adverse childhood experiences to become biologically ‘embedded’ by leaving genetic traces with life-long consequences (Aristizabal et al., 2020).

Many human studies have tried to identify the DNA methylation signatures associated with early exposure to adverse events in peripheral tissues such as blood or buccal cells (Cecil et al., 2020). As the functional impact of these changes has rarely been explored, however, it remains unclear whether they can impact future responses to stimuli. Overall, the extent to which DNA methylation affects gene expression is still poorly understood. Some studies report that methylation marks disrupt transcription factor binding and other gene expression mechanisms, but other evidence indicates they may have no effect on the activity of most regulatory elements (Kreibich et al., 2023). These conflicting results highlight the need to closely examine which type of impact methylation may have on various regulatory sites. Now, in eLife, Rachel Johnston, Jenny Tung and colleagues at Duke University and other institutes in Canada, Germany and the United States report having used a technique called mSTARR-seq to investigate the effect of DNA methylation on the activity of millions of sequences across the human genome, particularly in response to environmental factors (Johnston et al., 2024).

The team had previously developed this approach to assess the functional effects of DNA methylation on a large number of sequences ‘in one go’, but they had not examined then the impact of external stimuli. In their latest study, they aimed to address this gap by first using mSTARR-seq to assess the activity on a genome-wide level, including the most studied methylation sites in both their methylated and unmethylated states. This revealed that differences in methylation status impacted the activity of almost half of known regulatory regions, suggesting a genome-wide role of DNA methylation in gene regulation.

Next, Johnston et al. assessed whether pre-existing DNA methylation status influenced how cells respond to stimuli. To do so, they applied mSTARR-seq to immune cells exposed to molecules known to modulate inflammation and mediate stress responses. By modelling immune system activation and stress responses, the team highlighted thousands of regulatory regions that respond differentially to the compounds depending on their initial DNA methylation status. Further experiments using macrophages from human donors confirmed that differences in pre-existing methylation patterns predicted responses to viral infection (Figure 1).

**Figure 1.**
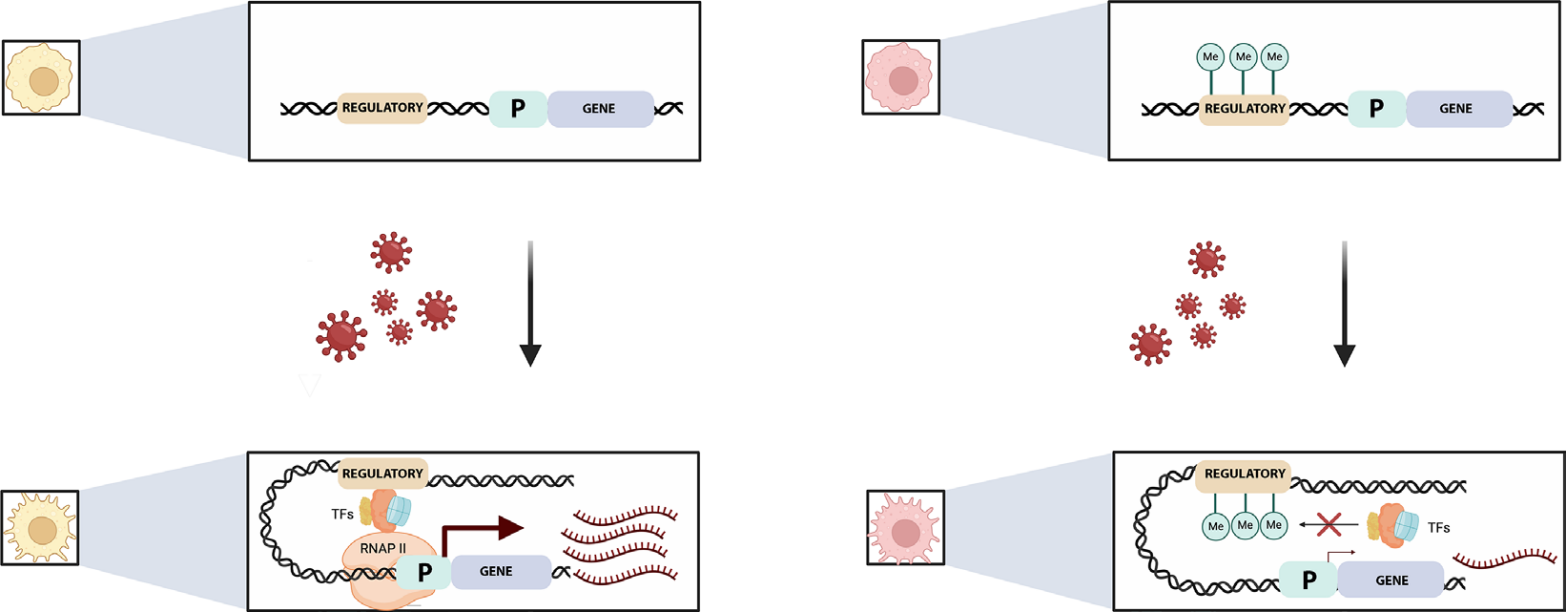
Pre-existing DNA methylation influences how macrophages respond to infection. Viral exposure activates macrophages and leads to changes in their gene expression. Johnston et al. show that the methylation status of certain sequences prior to infection can alter this response. For example (top left), an unmethylated regulatory element (brown) controlling a gene (blue) and its associated promotor (‘P’; green) is free to recruit transcription factors (TFs) after viral exposure (bottom left). This helps a complex known as RNAPII to bind to the promotor, allowing transcription to start (maroon arrow) and large numbers of mRNA transcripts to be produced (maroon). If the regulatory element is methylated, however (top right), the methylated groups (green circles labelled Me) interfere with the recruitment of the transcription factors (red cross, bottom right) and therefore with the binding of RNAP II. This impairs transcription and the associated production of mRNA transcripts.

Finally, the team explored whether methylation changes linked to early life stressors could influence gene regulation. To do so, they used data from 27 studies and compiled a list of genomic regions whose methylation levels are associated with adverse childhood events. Except for one study, these sequences were not more likely to be gene regulatory regions compared to chance; they were also not enriched in sites that the mSTARR-seq analyses highlighted as displaying DNA-methylation-dependent activity. This led Johnston et al. to suggest that DNA methylation marks linked to childhood adversity in peripheral tissues might serve as indicators of exposure to early stressors, rather than cause gene expression changes with long-lasting effects.

It is important to note, however, that these findings were established by comparing data from different cell types – the mSTARR-seq analyses are based on immune cell lines, while the studies examining DNA methylation and early stressors used blood, saliva or buccal samples. Yet the physical and psychological outcomes associated with early adverse experiences involve many tissues, which are known to present different methylation patterns (Eriksson et al., 2014; Rahman and McGowan, 2022). It is therefore possible that these marks have a more causal role in other cell types. Further studies investigating how DNA methylation impacts gene expression across tissues would help to clarify the connection between stress in early life, epigenetic changes, and later health outcomes.

Our interaction with our environment is not a passive process – not even at the level of gene expression. The work by Johnston et al. helps to dissect the complexity of this relationship, highlighting how DNA methylation modulates our response to external stimuli. Their study also suggests that we should carefully interpret the role this epigenetic process plays in the long-term impact of early stressors. Numerous questions remain, including about how these findings could be translated into new targets for preventing and treating disease.
